# Resistance pattern of *Acinetobacter* spp. isolated from various clinical samples in and around Kanchipuram

**DOI:** 10.1186/1471-2334-12-S1-P57

**Published:** 2012-05-04

**Authors:** S Senthamarai, S Sivasankari, C Anitha, V Venugopal, SK Amshavathani

**Affiliations:** 1 Dept of Microbiology, Meenakshi Medical College and Research Institute, Kanchipuram, Tamil Nadu, India

## Background

For the past two decades *Acinetobacter spp*. has been emerged as an important pathogen in various infections. Emergence of multi drug resistance limits the therapeutic option. So this study is aimed to determine the antibiotic resistance pattern of *Acinetobacter spp*. from various clinical samples.

## Methods

The study included a total of 1516 samples of which 892 were from pus and 624 were from sputum collected from patients at Meenakshi Medical College and Research Institute, Kanchipuram from Nov 2010 to August 2011. Samples were processed and identified according to standard protocol. The *Acinetobacter spp*. isolates were tested for antibiotic resistance against 16 antibiotics by Kirby Bauer disc diffusion method (CLSI guidelines).

## Results

Out of 1516 samples collected, 50 (3.3%) *Acinetobacter spp.* was isolated. Among which 35/50 (70%) were from pus, 15/50 (30%) were from sputum. All the isolates were found to be 100% resistant to ampicillin, 76% resistance to co-trimoxazole cefuraxime and ceftazidime, 64% to doxycycline. Ciprofloxacin and gentamicin showed 58% resistance, followed by netilmicin, amikacin and tetracycline with 57%, 50% and 46% resistance. Ofloxacin, sparfloxacin and levofloxacin showed 33%, 21% and 17% resistance. Least resistance was observed for amoxyclav, ceftriaxone/tazobactam and imipenem with 11%, 5% and 5% resistance respectively. Out of 50 isolates 31 (62%) were found to be multi drug resistant.

## Conclusion

Multi drug resistance among *Acinetobacter spp*. is of great clinical importance which provacates the need to screen and formulate appropriate antibiotic policy for the hospitals and prevent further development and spread of resistant strains.

